# Viral complementation allows HIV-1 replication without integration

**DOI:** 10.1186/1742-4690-5-60

**Published:** 2008-07-09

**Authors:** Huub C Gelderblom, Dimitrios N Vatakis, Sean A Burke, Steven D Lawrie, Gregory C Bristol, David N Levy

**Affiliations:** 1 Department of Basic Sciences and Craniofacial Biology, New York University College of Dentistry, New York, NY, USA; 2 Department of Medicine, David Geffen School of Medicine, University of California Los Angeles, Los Angeles, CA, USA

## Abstract

**Background:**

The integration of HIV-1 DNA into cellular chromatin is required for high levels of viral gene expression and for the production of new virions. However, the majority of HIV-1 DNA remains unintegrated and is generally considered a replicative dead-end. A limited amount of early gene expression from unintegrated DNA has been reported, but viral replication does not proceed further in cells which contain only unintegrated DNA. Multiple infection of cells is common, and cells that are productively infected with an integrated provirus frequently also contain unintegrated HIV-1 DNA. Here we examine the influence of an integrated provirus on unintegrated HIV-1 DNA (uDNA).

**Results:**

We employed reporter viruses and quantitative real time PCR to examine gene expression and virus replication during coinfection with integrating and non-integrating HIV-1. Most cells which contained only uDNA displayed no detected expression from fluorescent reporter genes inserted into early (Rev-independent) and late (Rev-dependent) locations in the HIV-1 genome. Coinfection with an integrated provirus resulted in a several fold increase in the number of cells displaying uDNA early gene expression and efficiently drove uDNA into late gene expression. We found that coinfection generates virions which package and deliver uDNA-derived genomes into cells; in this way uDNA completes its replication cycle by viral complementation. uDNA-derived genomes undergo recombination with the integrated provirus-derived genomes during second round infection.

**Conclusion:**

This novel mode of retroviral replication allows survival of viruses which would otherwise be lost because of a failure to integrate, amplifies the effective amount of cellular coinfection, increases the replicating HIV-1 gene pool, and enhances the opportunity for diversification through errors of polymerization and recombination.

## Background

Integration of the retroviral cDNA into cellular chromatin is a basic feature of retroviral replication [1-3] and is mediated by the viral integrase enzyme, a product of the pol gene whose substrate is a linear form of viral DNA. Chromatin supports the high levels of gene expression necessary for production of new virions and completion of the viral life cycle. Integration also ensures that the viral genome will persist for the life of the cell. When viral latency follows integration, one consequence is viral persistence in the face of highly suppressive antiviral therapy [4,5]. The integration process provides a target for the development of antiviral drugs [6].

When integration fails, virus replication is thought to be irretrievably lost, since unintegrated DNA (uDNA) by itself evidently does not support the level of gene expression necessary for virus production [7-11]. Thus uDNA is considered a replicative dead-end. Interestingly, *in vivo *and in cell culture HIV-1 integration has a very high failure rate, resulting in the accumulation of up to two orders of magnitude more uDNA than integrated viral DNA (iDNA) within cells [12]. HIV-1 infection *in vitro *[13-18] and *in vivo *[5,12,14,19-26] results in an abundance of uDNA regardless of cell type and activation status. In blood [5,19,20], lymphoid tissue [5,12] and brain [25,26] uDNA is up to 100 times more abundant than integrated DNA (iDNA). uDNA accumulates in both activated and quiescent T lymphocytes [5,14,19], in monocytes [21] and in patients with high or low viral loads [22,23]. HIV-1 DNA *in vivo *has been described as primarily a stable extrachromasomal episome [19,24]. Since reverse transcription destroys the RNA template, each copy of uDNA and iDNA represents a unique infection event with a potentially divergent genetic sequence.

Both linear and circular forms of uDNA accumulate in cells [27-29]. Linear DNA is quickly degraded within dividing T cells, with a half-life of less than one day [30], but is more stable in resting T cells [31], macrophages [32] and other non-dividing cells [33]. On the other hand, circular uDNA is highly stable and evidently lost only through cell death or dilution during cell division [30,34,35]. Importantly, both linear and circular uDNA are transcriptionally active [36]. Gene expression from uDNA has generally been investigated through the use of integrase inhibitor drugs or using viruses with mutations in the catalytic domain of integrase (Class I mutations) which prevent HIV-1 integration while preserving the ability of the virus to enter cells, uncoat and perform reverse transcription.

HIV-1 gene expression is divided into early and late phases, and only early protein products Tat [8,11,37] and Nef [32,37] have been detected in cells containing only uDNA. Late gene expression, requiring accumulation of sufficient Rev to direct the export of singly spliced and unspliced mRNA from the nucleus, does not occur at high enough a level to allow for virion production from uDNA [37,38]. Pre-integration DNA in activated T cells and transformed T cell lines expresses both early and late RNA, though only Tat and Nef are apparently translated [38]. Prior to integration in resting T cells, uDNA can express Tat and Nef proteins at levels which promote cell activation and enhance productive infection, suggesting a potential role in viral replication [37]. In macrophages, uDNA can remain transcriptionally active for at least 30 days [32]. Studies employing bulk analysis of cells have shown that uDNA is responsive to Tat activation [39]. Additionally, Vpr protein brought into cells within viruses increases uDNA gene expression of both Tat and Nef [39,40].

Multiple infection of cells is easily demonstrated in culture [16-18,41-44] and increases over time through reinfection [17,43,45-47]. HIV-1 entry apparently favors multiple infection, whether through cell-free virus or cell to cell contact [41,42]. Our previous work has shown that over several rounds of virus replication in culture or in human lymphoid tissue in an animal host, multiple infection proceeds with no observable inhibition [43]. The outcome is frequent coinfection and a high rate of recombination [43]. Within lymphoid tissues, particularly in germinal centers, cells are in close proximity, cognate and non-cognate immune cell contact is ongoing and the local concentration of virus is much higher than in the blood [48] favoring multiple infection [49]. Most striking, a study employing *in situ *analysis of splenocytes from HIV-1+ individuals observed an average of 3–4 proviruses per infected cell, with up to 8 in some cells [50]. Analysis of the genetic diversity within individual splenocytes could only be explained by additional unintegrated DNA [28]. More recently, Mattapallil et al. demonstrated that acute SIV infection results in an average of 1.5 proviruses per cell [51]. In cell culture experiments, infection at a frequency of 3 integrated proviruses per cell resulted in the accumulation of 10 times that amount of uDNA within the cells [16]. A convincing mechanism for abundant coinfection has been provided by recent descriptions of the viral synapse, in which contact between infected and uninfected cells leads to a directed delivery of high numbers of virions into cells [52-54].

Together, the documentation of uDNA gene expression and the presence of uDNA and iDNA together in cells suggests that these two forms of HIV-1 genomes might functionally interact. Here we investigated the influence of an integrated provirus on the activity and replication of viruses which fail to integrate.

## Results

### Single cell analysis of uDNA-directed gene expression

Gene expression from unintegrated DNA has been studied through bulk analysis of infected cells (RNA assays, western blot, etc.) and reporter viruses that employ luciferase, which is measured on bulk populations of cells [11,39,40]. Single cell analysis, on the other hand, would provide information on both the level of gene expression in individual cells and the number or proportion of cells containing genetically active uDNA. In order to perform single cell analysis we employed HIV-1 reporter viruses containing genes for the fluorescent proteins enhanced GFP, enhanced YFP, enhanced CFP and DsRedExpress ("DsRedX") [43,55]. GFP, YFP and CFP versions of these viruses have been previously described [43,55] and contain the reporter gene inserted immediately downstream of the envelope gene in the position normally occupied by the early gene nef, which is expressed from an IRES element. Schematic diagrams of each reporter virus are presented in [Additional file 1]. To study gene expression and virus replication from uDNA, we applied the diketo acid integrase inhibitor 118-D-24 [56,57] to cells at the time of infection, or we introduced a D116N Class I active site mutation [7,11] into the integrase gene of the reporter viruses. For single round replication experiments we employed envelope mutant variants which were pseudotyped with VSV-G protein [43,55].

**Figure 1:**
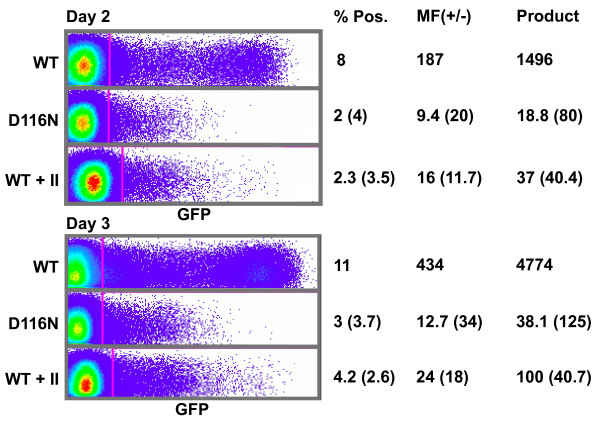
**Gene expression from cells infected with integrating and non-integrating HIV-1 reporter viruses**. NLENG1-ES-IRES [43] ("WT-GFP") or an integrase D116N mutant version of NLENG1-ES-IRES ("D116N-GFP"), each pseudotyped with VSV-G protein, were used to infect Jurkat cells (4.4 ng p24 on 2 × 10^5 cells) and analyzed by flow cytometry 48 and 72 hours after infection. Integrase inhibitor 118-D-24, or DMSO carrier, was applied to some cells at 200 μM at the time of infection with integrase-WT virus. Data are representative of several independent experiments. Table shows the percentage of cells that were GFP+ ("% Pos.") and the mean fluorescence (MF) of the GFP+ cells divided by the background fluorescence of the GFP-negative cells ("MF(+/-)"). "Product" is the % Pos. multiplied by the MF(+/-) and represents the overall GFP gene expression from the infected cells. Numbers in parentheses represent the fold reduction vs. WT virus. "II" = integrase inhibitor. Dot plots show GFP fluorescence in the X axis and arbitrarily chosen red background fluorescence on the Y-axis.

We first compared early HIV-1 gene expression in T cells from integrating and non-integrating viruses, focusing on the period corresponding to the average *in vivo *life span of an infected T cell. We infected cells at a low MOI (≤0.12) to limit the amount of multiple infection, then analyzed the cells by flow cytometry at 48 and 72 hours after infection. Prior bulk analyses of cells have shown that uDNA expression from early HIV-1 genes is about 2 orders of magnitude lower than from an integrated provirus [11,39,40]. Our single cell analysis showed reductions compared to WT virus in both the number of fluorescent cells and the level of fluorescence in each cell which were similar for both the integrase mutation and the integrase inhibitor (Figure 1). The number of fluorescent cells was reduced 2.6 to 4 fold vs. WT virus in untreated cells (up to 10 fold reduction was observed in some experiments), while the level of the reporter expression in fluorescent cells was reduced 11.7 to 34 fold. The cumulative effect of fewer cells with active viruses and lower expression per cell yields an effective reduction in gene expression of 40–125 fold for uDNA vs. integrating virus, a result that is consistent with the previous bulk analyses. Integrase inhibitor slightly increased background autofluorescence from the cells. This is apparent in the rightward shift of the large GFP-negative population of cells treated with integrase inhibitor in Figure 1. Cells that are not exposed to virus show the same shift in response to integrase inhibitor (not shown). However the mean fluorescence of the GFP+ cells treated with integrase inhibitor was higher relative to background than the cells infected with the integrase mutant. All of this additional fluorescence was found in a minor population of cells that were as bright as the cells infected with WT virus, suggesting that integrase inhibitor had failed to prevent integration in a small minority of cells.

Interestingly, the level of gene expression increased from day 2 to day 3, as did the percentage of cells that were fluorescent, for both the integrating virus and the non-integrating viruses. The DNA of an integrated virus will be duplicated and partitioned to each daughter cell, while uDNA will be diluted by cell division in this rapidly dividing population. Therefore the increase in uDNA gene expression from day 2 to day 3 indicates that rather than being rapidly inactivated or only active prior to viral integration in these cells, uDNA is a source of early HIV-1 gene expression throughout the average ≤2 day life span of *in vivo *infected T cells.

### Most uDNA is inactive by itself but is subject to activation by an integrated provirus

**Figure 2:**
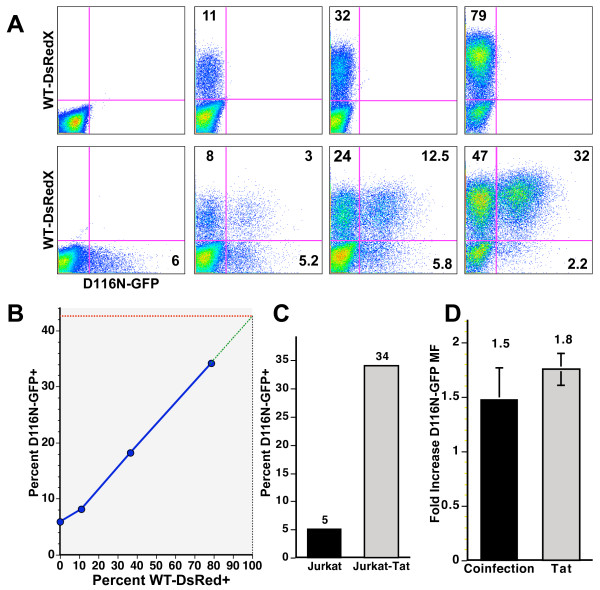
**Activation of uDNA gene expression by coinfection with integrating virus**. **A**. Upper panels: 2 × 10^5 Jurkat cells were left uninfected (left panel) or infected with increasing amounts of WT-DsRedX virus (5, 20, 60 ng p24 respectively). Lower panels: Cells were simultaneously infected with equal amounts of D116N-GFP virus (10 ng p24) and the same amount of WT-DsRedX virus as in the panel directly above. Numbers represent the percentage of cells in each indicated quadrant. All viruses were envelope-defective and pseudotyped with VSV-G protein to limit viruses to a single round of replication. **B**. The solid blue line plots the percentage of cells that express D116N-associated GFP fluorescence from the lower panels in A (Y axis) as a function of the amount of WT-DsRedX infection (X axis). Extrapolation to 100% infection with WT-DsRedX virus implies that 42% of cells are infected with a D116N-GFP virus that is capable of generating fluorescence in the presence of a coinfecting integrating virus (dashed lines). **C**. Effect of Tat on the percentage of cells expressing D116N-associated GFP fluorescence. As in Figure 2A-B, viruses were envelope defective and pseudotyped with VSV-G to overcome differences in CD4 levels on Jurkat and Jurkat-Tat cells and to limit viruses to a single round of replication. qPCR for HIV-1 DNA found equal infection of Jurkat and Jurkat-Tat cells with 0.9 HIV-1 genomes/cell by real time DNA qPCR. A nearly 7-fold increase in the percentage of cells expressing D116N-associated GFP fluorescence is similar to results of coinfection in panel B and demonstrates that Tat is sufficient for the transactivation provided by coinfecting viruses. This experiment is representative of multiple independent experiments. **D**. The increase in mean fluorescence in Jurkat from D116N-GFP virus as a result of coinfection with an integrase-WT virus as a result of Tat transactivation in the Jurkat-Tat cell line. Coinfection data represent the mean fluorescence of cells coinfected with D116N-GFP and WT-DsRedX viruses divided by the mean fluorescence of cells infected with only D116N-GFP virus.  Data represent multiple samples from each of 3 independent experiments. Tat data represent the mean fluorescence of GFP+ Jurkat-Tat cells divided by the mean fluorescence of GFP+ Jurkat cells infected with D116N-GFP virus. The average and SD are derived from multiple samples of a representative experiment.

The co-residence of uDNA and iDNA in cells has been documented *in vivo *and in tissue culture [16,18,28]. To examine uDNA gene expression under this circumstance, we infected T cells with either a D116N-GFP mutant virus alone or simultaneously infected the cells with the same amount of mutant virus plus increasing amounts of a WT-DsRedX virus (Figure 2A). Each virus was envelope-defective and pseudotyped with VSV-G protein for single round infection. As more cells were infected with increasing amounts of integrating WT-DsRedX virus, more cells displayed gene expression from the integrase mutant D116N-GFP virus. Similar results are obtained by infecting cells sequentially with mutant and WT a day apart, indicating that this result is not due to viruses adhering to each other prior to entry (not shown). The percentages of cells showing gene expression from the D116N-GFP virus in response to coinfection is graphed in Figure 2B, where a linear response to increasing WT-DsRedX infection is evident. Extrapolation to 100% WT-DsRedX infection suggests a potential 7 fold increase in the number of cells displaying uDNA-driven GFP fluorescence.

The activation of uDNA is presumably through the effects of Tat provided by the integrated provirus. To test this we infected Jurkat and Jurkat-Tat cells, the latter cell line constitutively expressing Tat protein from an integrated plasmid [58]. Infection of these two cell lines with identical amounts of VSV-G pseudotyped virus (which bypasses differences in CD4 expression) resulted in a very similar outcome as coinfection between WT and D116N viruses, confirming the influence of Tat on the number of cells displaying uDNA-directed gene expression (Figure 2C). PCR analysis showed that Jurkat and Jurkat-Tat cells were infected with equal amounts of virus (see below).

The mean fluorescence intensity of cells infected with D116N-GFP virus was increased only an average of 1.5 fold in the coinfected cells compared with cells infected with just the integrase mutant, and Tat increased the brightness of D116N infected cells an average of 1.8 fold (Figure 2D). Thus the primary effect of coinfection and of Tat is to drive uDNA gene expression when it otherwise would not occur at levels measurable by GFP expression, rather than to dramatically increase the level of uDNA gene expression from viruses that are already active.

DNA qPCR analysis revealed an average of 0.9 uDNA HIV-1 genomes per cell ("DNA MOI" of 0.9) in both the Jurkat and J-Tat cells infected with the D116N virus. Jurkat cells infected with the D116N virus displayed GFP fluorescence in 5% of cells, which, by Poisson distribution analysis corresponds to a "fluorescence MOI" of 0.05, or 18.4 times less than the actual DNA-based MOI. This indicates that only a small fraction of uDNA genomes were active to a level detectable by flow cytometry. In the Jurkat-Tat cells, on the other hand, 34% of the cells were GFP+, which corresponds to a fluorescence MOI (by Poisson analysis) of 0.42, which is nearly half of the DNA MOI of 0.9 genomes/cell.

### Late gene expression from uDNA

In order to generate new virions, HIV-1 must transition to late gene expression by first expressing a threshold level of Rev protein, which then facilitates export of unspliced and singly spliced RNA to the cytoplasm. These Rev-dependent late RNA encode late accessory proteins Vpr, Vpu and Vif, and the structural polyproteins Gag, Pol and Env. Rev-dependent unspliced RNA molecules are also packaged into virions as viral genomes [38]. To study late gene expression from uDNA at the single cell level we created a dual early-late reporter virus by adding the gene for murine Heat Stable Antigen (HSA) (a.k.a. murine CD24) in the vpr region [59] to the WT-GFP and D116N-GFP viruses. HSA is a cell surface protein that can be detected with antibodies in flow cytometry. GFP is expressed from Rev-independent (early) mRNA, and HSA is expressed from Rev-dependent (late) vpr mRNA. Thus cells infected with this dual reporter virus will be GFP+HSA- single positive when the virus is in the early phase of HIV-1 gene expression, whereas cells containing a virus in the late Rev-dependent phase of gene expression will be GFP+HSA+ double positive. Since Vpr protein brought into cells by infecting virions contributes to gene expression from uDNA [40], and the vpr gene is deleted in this virus, we generated Vpr+ virions by co-transfecting 293T cells with a Vpr expression plasmid. We observed Vpr enhancement uDNA gene expression up to 2 fold under these conditions (not shown).

**Figure 3:**
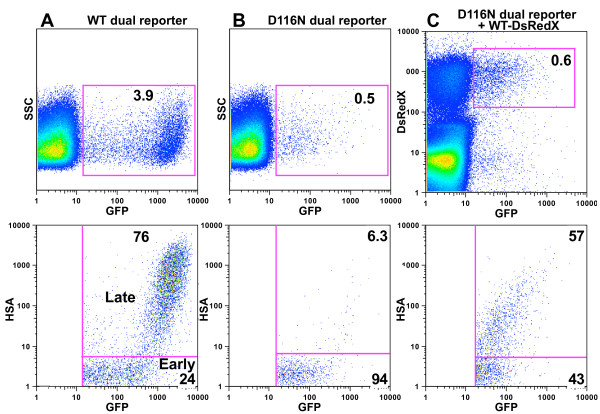
**Late HIV-1 gene expression from integrating and non-integrating HIV-1**. Activated primary T cells were infected with **A**. a WT-GFP virus, **B**. a D116N-GFP/HSA dual reporter virus or **C**. coinfected with the D116N-GFP/HSA dual reporter and a WT-DsRedX virus. Cells were analyzed by flow cytometry 48 hours after infection. Upper panels show all infected cells, total infection rates, and the gates applied for analysis in the lower panels. Lower panels show Rev-independent early gene expression (GFP) vs. Rev-dependent late gene expression (HSA). Gating is on the fluorescent cells in the top panels in order to highlight the ratio of cells displaying early (GFP+HSA- cells) to those exhibiting late HIV-1 expression (GFP+HSA+ cells). Data are representative of several independent experiments. Similar results are obtained with Jurkat cells.

Analysis of activated T cells infected with an integrase-WT dual reporter virus showed late gene expression within 50–85% of GFP+ cells 48 hours after infection (76% in this experiment is typical) (Figure 3A). The overall infection frequency was 3.9%. Late gene expression was almost entirely limited to the cells displaying the highest levels of GFP expression, indicating that during HIV-1 replication Rev does not down regulate early gene expression. Infection with equal amounts of an integrase mutant dual reporter virus resulted in 0.5% GFP+ cells, of which only 6.3% showed late gene expression, consistent with prior studies on bulk cell populations [37,38] (Figure 3B). To test the influence of a coinfecting integrating virus we simultaneously infected T cells with the D116N GFP/HSA dual reporter virus and a WT-DsRedX virus (which does not express HSA) and gated on the DsRedX+GFP+ double positive coinfected cells (Figure 3C). 57% of these coinfected cells displayed HSA expression from the integrase mutant, indicating an efficient switch to late gene expression by the uDNA. The result was a little lower than expression from the WT virus; however, since about one-quarter of WT viruses do not reach late gene expression at this time point, it is reasonable to assume that these viruses would be unable to boost a coinfecting virus to late gene expression, perhaps as a result of inadequate Rev expression. Indeed, PCR analysis of GFP+HSA- cells infected with integrase-WT virus showed that many of these cells contained integrated genomes yet had not transitioned to late gene expression (not shown).

### Completion of a full infectious virus life cycle by uDNA through viral complementation

**Figure 4:**
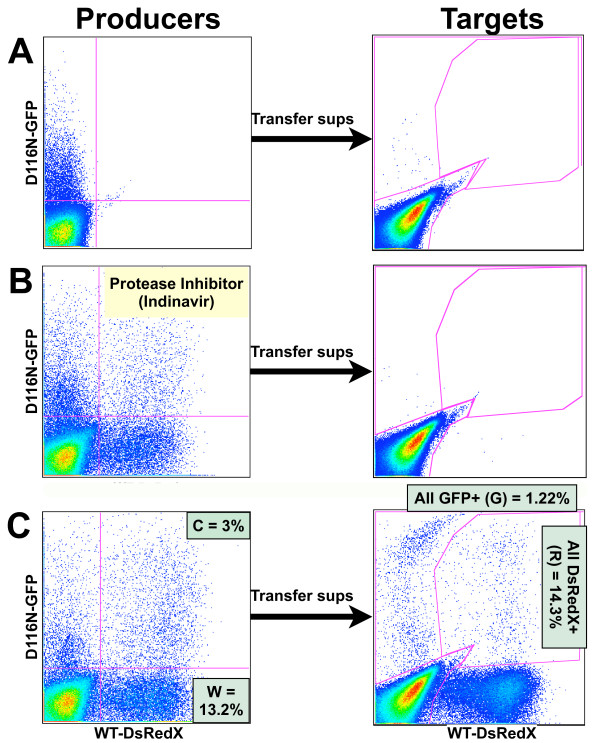
**Completion of the HIV-1 replication cycle by uDNA via coinfection with an integrating virus**. Jurkat cells were infected with D116N-GFP virus only, washed and incubated with protease to remove residual virus, then 2 days later the resulting culture supernatants were used to infect Jurkat-Tat target cells. **A**. Cells containing only uDNA show little infectious virus output. **B**. Cells that were coinfected with WT-DsRedX and D116N-GFP viruses and treated with HIV-1 protease inhibitor Indinavir show little virus output. **C**. Procedures followed as in B, except no Indinavir was present. Virus transfer to Jurkat-Tat target cells results in both DsRedX fluorescence and GFP fluorescence, indicating that infectious viruses were generated which package and deliver functional genomes derived from unintegrated D116N-GFP DNA within the producer cells. Data are representative of multiple independent experiments. "C = 3%", "W", "G", "R" refer to figure 5.

Late gene expression from uDNA begs an important question: Do virions generated by an integrated provirus package functional genomes derived from uDNA, allowing uDNA to complete its replication cycle and contribute to the replicating gene pool? To test this notion we performed a 2 cycle virus replication assay, where the first infection generates "producer" cells that are coinfected with uDNA and iDNA, and the second generation viruses from these producer cells are then assayed for their ability to deliver functional uDNA-derived genomes to "target" cells. For the remaining series of experiments we employed viruses containing functional envelope genes with no virus pseudotyping and infected cells via HIV-1 envelope mediated entry. We infected Jurkat T cells with D116N-GFP alone or a combination of WT-DsRedX and D116N-GFP HIV-1. One day after infection we washed these infected producer cells and treated them with the broad spectrum protease pronase in order to remove residual input virus, then placed the cells back in culture for 1 to 2 days to allow *de novo *virus production. These second generation viruses were used to infect Jurkat-Tat cells which were then analyzed by flow cytometry to assess the proportion of GFP+ and DsRedX+ cells. Jurkat-Tat cells were employed as targets in order to activate uDNA genomes which otherwise would be silent or express below the threshold of detection, thus providing a more reliable accounting of infection. Since integrase mutant viruses are used only as a convenient surrogate for "unlucky" but otherwise replication competent viruses which fail to integrate, the use of Tat-containing target cells improves the relevance of the analysis. As expected, producer cells infected with only the integrase mutant released very little to no infectious virus (Figure 4A). Supernatants from coinfected producer cells treated with an antiviral protease inhibitor (Indinavir) showed very little infectivity, demonstrating a lack of carryover of virus from the initial infection (Figure 4B). On the other hand, cells coinfected with WT-DsRedX and D116N-GFP generated infectious viruses which conferred GFP and DsRedX fluorescence to target cells (Figure 4C). Application of reverse transcriptase inhibitors (AZT and Efavirenz) to target cells prevented the appearance of fluorescence from both viruses (not shown), demonstrating that the appearance of fluorescence in target cells requires reverse transcription. In some experiments virus stocks were treated with DNase in order to eliminate possible plasmid contamination, with no effect on the experimental outcome (not shown). These results establish that uDNA-generated mRNA is packaged into virions and delivered as a functional replicating genome through complementation by an integrated provirus.

**Figure 5:**
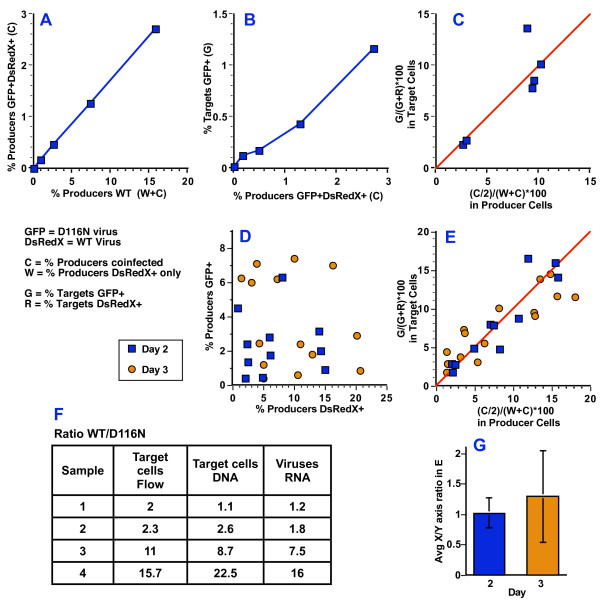
**Measurement of the efficiency of uDNA replication during coinfection**. **A**. The relationship between the infection of producer cells with WT-DsRedX virus and the presence of producer cells displaying fluorescence from both WT-DsRedX and D116N-GFP viruses. The same amount of D116N-GFP virus was used to infect each population of cells, yielding 4.5% GFP+ cells without addition of WT-DsRedX virus. As increasing amounts of WT-DsRedX virus are used to coinfect cells, the percentage of cells displaying both GFP and DsRedX fluorescence increased linearly, as shown. Data were collected day 2 after infection of producer cells, at the time of virus transfer to target cells. Values represent the percentage of producers that were double-positive (X axis) vs. the percentage of producers that were DsRedX+ (both single and double positive). **B**. The relationship between the frequency of double positive GFP+DsRedX+ producer cells and the frequency of the resulting GFP+ target cells. **C**. Relationship between the GFP+ producer cells and the ratio of D116N-GFP and WT-DsRedX viruses conferred to target cells. The X axis predicts the percentage of viruses generated by producer cells that will confer GFP to target cells. The formula for the X axis assumes equal production of D116N-GFP and WT-DsRedX viruses from double positive producer cells and production of only DsRedX viruses from DsRedX+ cells. The Y axis presents the percentage of all fluorescent target cells that are GFP+. In the target cells, all GFP+ cells (G) and DsRedX+ cells (R) are tallied, and cells which are double positive GFP+DsRedX+ are counted in both categories. The red line represents unity between the two formulas, where the assumptions used in the X axis formula are true. **D**. The experiment presented in A-C was repeated using primary activated CD4+ T cells as producers, and the percentage of D116N-GFP and WT-DsRedX in each producer cell population is shown to illustrate the wide range of MOI and WT/D116N ratios employed. Blue squares represent producer cells on day 2 after infection and orange circles represent producer cells on day 3 after infection. Data are aggregated from 3 independent experiments. **E**. The relationship between producer and target cells as in Figure 5C using primary T cell producer cells, showing data from the 3 independent experiments in Figure 5D. **F**. WT-DsRedX to D116N-GFP ratio in the target cells by flow cytometry and DNA PCR, and by RT-PCR on the viruses used to infect them. Numbers represent the ratio of DsRedX+ cells to GFP+ cells, or the ratio of WT-DsRedX to D116N-GFP nucleic acid in indicated samples. This experiment is representative of two independent experiments. **G**. Averages and standard deviations for the cumulative data in Figure 5E.

### Efficiency of replication of the uDNA-derived genomes

Next we performed a quantitative analysis of the contribution of uDNA-derived genomes to the replicating virus population. By comparing the ratio of GFP+ to DsRedX+ target cells to the GFP+/DsRedX+ ratio in the producer cells, the efficiency of viral packaging of functional uDNA genomes can be gauged. Our analysis is based on the following assumptions: Producer cells that are infected with only WT virus ("W" in Figures 4 and 5) will generate virions that have only WT genomes in them, while the cells infected with only the integrase mutant will generate little or no virus, and so are ignored in calculations. Cells that are coinfected with WT and D116N viruses ("C" in Figures 4 and 5) will generate a population of virions containing both WT and integrase mutant genomes at some unknown ratio. For example, if coinfected cells package 50% WT and 50% D116N genomes into viruses, then the output of D116N virions from coinfected cells ("C") is C/2. The percentage of D116N genomes in the total virus population from producer cells will be (C/2)/(W+C)*100. Target cells will be GFP+ and/or DsRedX+ in a proportion that reflects the ratio of virions containing functional genomes of either type. Although HIV-1 virions contain two RNA genomes, which can be derived from the same or different producer viruses, this diploidy can be ignored at present since virions will generate only a single cDNA in target cells and will confer either GFP or DsRedX fluorescence but not both.

In order to examine the influence of MOI or uDNA/iDNA ratio on the efficiency of complementation, we infected producer cells with a constant amount of D116N-GFP virus and increasing amounts of WT-DsRedX virus, then transferred viruses to target cells on day 2 after infection of producer cells (Figure 5A–C). As the amount of WT virus used to infect producer cells was increased (while holding the amount of mutant virus constant), the frequency of coinfected producer cells (C) showing uDNA gene expression rose (Figure 5A) (similar to the upper right quadrants in Figure 2A). As predicted, the number of viruses generated which deliver the mutant genome to target cells increased in direct proportion to the number of coinfected producer cells (Figure 5B).

If WT and integrase mutant viruses generate equal amounts of virion-packaged genomes in the coinfected cells, then the ratio of GFP to DsRedX in the target cells should reflect the following: (C/2)/(W+C) in the producer cells = G/(G+R) in the target cells. Unity in this relationship is represented by the red line in Figure 5C. Surprisingly, at each amount of input virus and ratio of WT-DsRedX to D116N-GFP viruses used to infect producer cells, the ratio of GFP to DsRedX in target cells adhered closely to this value. WT-GFP and D116N-DsRedX viruses produced identical results (not shown). Virus containing another Class I mutation, D64E, generated identical results as the D116N mutant (not shown).

We next tested this relationship using primary activated CD4+ T cells as producer cells, transferring virus to target cells on both day 2 and day 3 after infection of the producer cells. After day 3, re-infection of producer cells with second round viruses would obscure meaningful results regarding the ratio of uDNA and iDNA genome production, so no attempt was made to carry the experiment past day 3. We infected the producer T cells at a wide range of MOI (0.004 to 0.063 D116N-GFP, 0.007 to 0.23 WT-DsRedX based on the percentage of cells fluorescent in each color by a Poisson distribution formula, and a ratio of GFP+ to DsRedX+ cells from 0.16 to 24.31) (Figure 5D). As seen in Figures 5E and 5G, the resulting ratios of GFP+/DsRedX+ target cells adhered well to the rule generated in Jurkat cells.

To further validate our flow cytometry analysis, we measured by quantitative real time PCR the GFP and DsRedX DNA products of reverse transcription in 4 samples of Jurkat-Tat target cells with widely varying infection rates and GFP+/DsRedX+ ratios. We also analyzed by RT-PCR the DsRedX and GFP genomes within the viruses used to infect these target cells. Both the ratio of DsRedX to GFP DNA in the target cells and the RNA content of the viruses reflected very closely the proportion of cells displaying GFP and DsRedX fluorescence, confirming that flow analysis of target cells accurately describes the packaging efficiency of the genomes (Figure 5F).

### Phenotypic complementation of a genetic defect during productive coinfection

**Figure 6:**
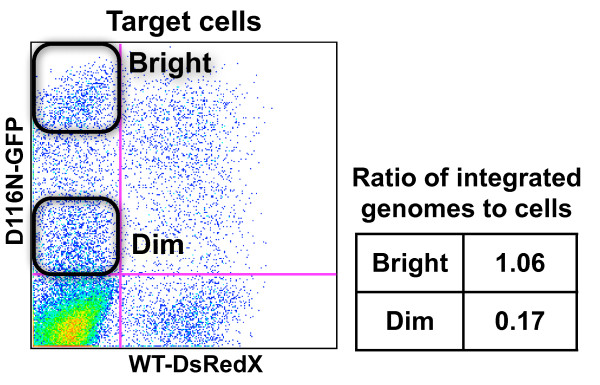
**Phenotypic complementation between WT and D116N in virions**. Target cells from virus transfer experiment in Figure 4. Two days after infection of target cells bright and dim GFP+ cells were sorted by FACS, then qPCR for integrated DNA was performed on the sorted cells. The integration of D116N-GFP DNA demonstrates that integrase mutant genomes are complemented by WT integrase within virions.

Multiple infection of cells provides the opportunity for phenotypic complementation through the mixing of proteins in virions. A defective gene in one virus may be complemented by a functional gene in another virus, thus allowing less fit viruses to persist. HIV-1 may even evolve towards lower fitness as a result of multiple infection of cells while still causing disease [60]. In our virus transfer experiments, typically one half of the GFP+ target cells displayed fluorescence as bright as that generated by WT viruses (compare, for example the relative absence of bright GFP fluorescence in producer with the cluster of bright GFP+ target cells in Figure 4C). The appearance of bright GFP+ target cells suggests that the D116N-GFP viral genomes had undergone integration in the target cells. Successful integration by the integrase mutant cDNA would most likely result from packaging of WT integrase together with the integrase mutant RNA. To test for integration we sorted the GFP bright and GFP dim populations of the single color target cells and analyzed the cells by real time PCR [13] for integrated and viral DNA (Figure 6). Among the sorted GFP+DsRedX- bright target cells there were 1.06 integrated genomes per cell, while within the GFP+DsRedX- dim cells there were only 0.17 integrated genomes per cell. This demonstrates that viruses with essentially zero fitness can persist through phenotypic complementation of a specific defect by a coinfecting virus.

### Recombination between uDNA-derived and iDNA-derived genomes

**Figure 7:**
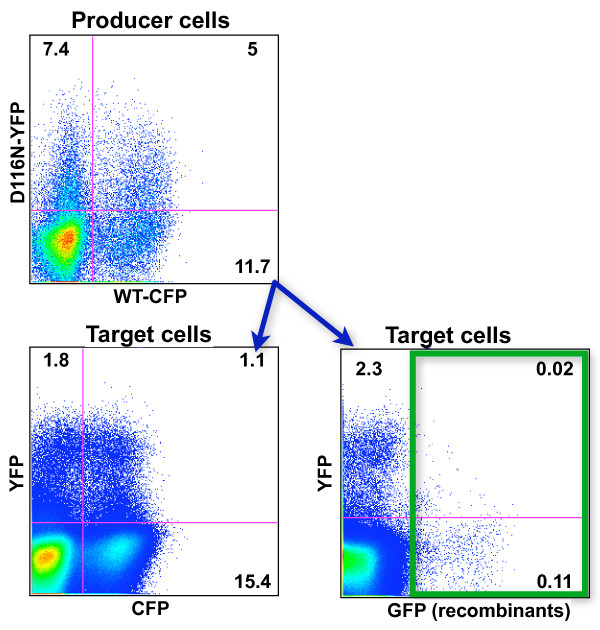
**Recombination between uDNA-derived and iDNA-derived genomes**. Jurkat cells were simultaneously infected with D116N-YFP and WT-CFP viruses and a virus transfer experiment performed as in Figure 4. The appearance of GFP+ target cells is strictly dependent upon copackaging of YFP and CFP genomes into virions during virus assembly in producer cells and reverse transcription during second round infection of target cells [43]. Numbers represent the percentage of cells in each quadrant. Data are representative of multiple independent experiments.

Recombination occurs during infection when reverse transcriptase switches templates and generates a cDNA that is a mosaic of the two co-packaged RNA genomes. Recombination only occurs between genomes that are co-packaged into virions, and does not occur during infection of cells with two different but homozygous viruses. Thus the appearance of recombinant viruses is a definitive indication that two different genomes have been packaged into single virions, infected new cells and undergone reverse transcription. We developed an experimental system that allows study of retroviral recombination on any cell type using flow cytometry [43]. In this system, recombination within the YFP and CFP genes in reporter viruses generates a novel sequence that confers GFP fluorescence to target cells. To test recombination between uDNA-derived and WT-derived genomes, we infected producer cells with D116N-YFP and WT-CFP viruses, then analyzed target cells for CFP, YFP and GFP fluorescence. As seen in Figure 7, GFP+ target cells appeared, demonstrating recombination between uDNA-derived and iDNA-derived genomes.

## Discussion

Here we show that unintegrated HIV-1 can participate in the replication of HIV-1 and contribute to its diversification. We found that when alone in cells, a small fraction of unintegrated HIV-1 DNA showed spontaneous gene expression measured by GFP fluorescence, but that through complementation with Tat from a coinfecting integrated provirus, a substantial fraction of uDNA was activated. During coinfection uDNA generated RNA genomes that were packaged into virions and delivered into new cells, where they underwent reverse transcription, recombination and integration. Although HIV-1 uDNA is not a strong template for gene expression, uDNA-derived genomes were nevertheless represented at a substantial level in the replicating pool of viruses, suggesting that replication of unintegrated HIV-1 may be biologically significant.

These findings have broad implications for the replication, diversification and persistence of HIV-1. Most fundamentally, viral sequences that were once believed to be lost owing to a failure to integrate may in fact be rescued by viral complementation. Since each copy of uDNA and iDNA represents a unique infection event with a potentially divergent virus, the rescue of unintegrated viruses would function to preserve extant viral diversity and lessen the chance that genetic information is lost through random drift. An important parameter in the evolutionary dynamics of HIV-1, the effective population size [61-67], defined as the number of individuals in a population which contribute to the next generation, could be substantially amplified through this mechanism. By rescuing viruses which would otherwise be lost, complementation of uDNA greatly increases the *effective *amount of cellular coinfection. Coinfection is the prerequisite for the copackaging of divergent genomes, and copackaging is the prerequisite for recombination, so the opportunity for recombination is enhanced. This observation provide a mechanism to explain *in situ *analysis of splenocytes of HIV-1 infected individuals showing frequent multiple infection with co-residence of uDNA and iDNA and extraordinarily and inexplicably high recombination [50]. Additionally, since each copy of uDNA and iDNA represents a unique reverse transcription event with the highly error-prone viral polymerase, the effective amount of mutation is increased by the preservation of unintegrated viruses. In conclusion, what has been thought to be a "bug" in the replication program of HIV-1 (the frequent failure to integrate) might now be seen as a "feature" which amplifies viral diversification and promotes survival of the quasispecies though cooperative interactions among its members [64,67,68].

The reasons for failure to integrate are not well understood, but there is likely to be a large stochastic component, where integrase may simply fail to find a chromatin target [27]. The proliferation of uDNA is thus not necessarily an indication of inherent defects among the unintegrated viruses, but rather this population probably reflects the replicating virus population as a whole. Other factors contributing to failure to integrate include possible degradation of linear DNA ends by cellular exonucleases before integration can occur, preventing integrase-mediated ligation of the viral DNA into the host chromosome. The preintegration complex (PIC), comprising the viral cDNA and several viral proteins including integrase, must be preserved intact for integration to succeed. The PIC has a limited functional life span [69] and can be inactivated by cellular proteases [70], rendering the viral DNA vulnerable to attack by cellular defense mechanisms, including nucleases and other enzymes which modify foreign nucleic acids. Whether uDNA as a whole contains more cell-induced damage than iDNA remains to be investigated.

The 3 most common forms of uDNA are linear, 1-LTR circles and 2-LTR circles [27-29]. Linear DNA predominates *in vivo *and *in vitro*, with a ratio of linear, 1-LTR circles and 2-LTR circles of about 20:9:1, respectively [71-73]. *In vivo *most linear uDNA molecules lack the blunt ends necessary for integration [5]. Linear DNA can become circularized by cellular repair mechanisms [1,74,75] within the cell nucleus; thus the presence of HIV-1 circles is a positive indication of nuclear import. We have not characterized which species of uDNA contribute to the replicating pool, but there is no *a priori *reason why both linear and circular uDNA could not generate full length genomes for virion packaging. All classes of linear and circular uDNA are probably transcriptionally active, with 1-LTR circles being more active than 2-LTR circles [36]. Both integrase inhibitors and active site integrase mutant HIV-1 generate excess 2-LTR circular forms compared with WT virus for reasons that are incompletely understood [8,38], so it is possible that if 2-LTR circles are particularly efficient templates for generating packaged genomes, our experimental system could exaggerate the contribution of uDNA to viral replication. This does not seem likely, since linear uDNA predominates over circles. Even though linear uDNA is quite labile and is lost within a day or two after infection of activated T cells, the half life of infected T cells *in vivo *is of similar length; thus all forms of uDNA, linear and circular, will be present throughout the life of the infected T cell *in vivo*.

Whether integrated or not, the HIV-1 promoter is capable of limited transcription prior to the production of Tat, the viral transactivating protein [76]. This pre-Tat transcription is necessary to initiate HIV-1 expression, and in the case of the integrated provirus, to complete the replication cycle. Once initial Tat is produced from an integrated provirus, a positive feedback loop is initiated that increases HIV-1 expression by about 3 orders of magnitude. In the case of uDNA, the sensitivity of the HIV-1 promoter to Tat transactivation is reduced dramatically in comparison with the integrated provirus, and the virus does not reproduce. The reasons for the low expression from uDNA are not well understood at the mechanistic level, but as protection from intracellular parasites it is clearly to the cell's advantage to favor transcription from chromatin rather than from extrachromasomal nuclear DNA. We observe about a 10 fold increase in total uDNA gene expression in response to Tat. However, our single cell analysis reveals that the majority of this increase is the result of activation of previously latent genomes, seen as an increase in the number of cells displaying uDNA gene expression, rather than an activation of uDNA to the high level of expression achieved by iDNA.

Gene expression from uDNA might generate proteins that are functionally or antigenically different from those expressed from the integrated provirus, with several possible consequences. We have shown that phenotypic complementation occurs between integrated and non-integrated viruses, through the packaging and delivery of uDNA-derived genomes into target cells and the integration of these genomes into target cells. We observed a high frequency of integration by integrase mutant viruses in the second round of replication, which must result from complementation by WT integrase. It also seems likely that the unintegrated virus could complement functions of the integrated virus, though this awaits further study.

A coinfecting virus may positively influence the replication of a second virus (as we have shown) or alternatively it might negatively influence another virus within the same cell. Whether viral interactions are positive or negative will depend on several factors. It seems possible or even likely that coinfecting viruses may compete for cellular resources such as transcription factors or other cellular components essential for virus expression, transport and assembly. In addition, dominant negative variants of viral proteins could inhibit replication of all viruses within a cell [77]. It might be useful to exploit such an interfering mechanism to therapeutically intervene in HIV-1 infection [78] with non-integrating lentiviral vectors [79,80]. Such vectors might be able to persist for some useful length of time through the coinfection complementation mechanism we describe here, as a form of vector mobilization [81,82]. An obvious potential disadvantage to replication of non-integrating vectors would be unintended recombination with the "native" viruses.

Another potential consequence of uDNA gene expression might be expansion of the repertoire of HIV-1 antigens presented to the immune system [10,28,50]. Our findings support this possibility and point to the prospect that through uDNA activation during coinfection, cells may more frequently present a diverse collection of epitopes than previously considered.

The high proportion of inactive but rescuable uDNA genomes makes it tempting to speculate that uDNA may constitute a reservoir of latent virus in cells that can be recruited into the replicating virus population by reinfection. In fact, circular forms of uDNA are highly stable and are only lost through dilution during cell division or by death of the infected cell [30,34,35]. Since macrophages and resting T cells do not replicate, they could provide an ideal environment for such a reservoir that has the potential to be substantially larger than the documented reservoir of integrated proviruses within resting T cells [5,83]. In this context it is important to note that gene expression from uDNA is substantially higher in non-proliferating cells than in actively replicating cells [27]. In addition, a recent study has shown that both linear and circular uDNA can persist in macrophages for at least 30 days [32]. Elegant studies from the Siliciano group and others have described a form of pre-integration latency, where activation of resting cells soon after infection allows virus integration and productive infection to proceed [69,84]. This form of latency lasts only a day or two and depends upon the PIC remaining functional so that it can mediate viral integration. In contrast, the form of latency we propose depends only on the uDNA remaining competent for transcriptional activation by an incoming virus.

To limit the generation of uDNA by the WT viruses, we kept infection rates to the lowest levels feasible, frequently employing an MOI of ≤0.05. However, we have not quantified how much uDNA was generated by the integrase-WT viruses, nor do our calculations consider possible uDNA generated by the WT viruses. In the virus transfer experiments (Figure 5) we employed a wide range of MOI and ratios of WT to D116N in part to control for this variable. For the most part the packaging efficiency of the D116N genomes was independent of the ratio of WT to D116N viruses used to infect producer cells and was directly linked with the appearance of doubly infected producer cells. A partial exception to this rule was virus generated day 3 after infection of primary T cell producers, where there was some reduction in D116N replication when a high ratio of D116N to WT was employed (Figure 5E). The variability of the data also increased considerably between days 2 and 3, though there was no statistical significance between the aggregate results from each day. By day 3 activated T cells will have divided up to two times, diluting the D116N uDNA genomes but not the integrated proviruses, which replicate with the cellular DNA. This effect seems to be more apparent in cells with a high D116N copy number. A more thorough parsing of these relationships is currently underway.

This study and prior studies observe that uDNA is never as strong a template for gene expression as iDNA. Yet uDNA is able to successfully compete with the integrated provirus for packaging into infectious virions. In fact, uDNA seems to be as efficient as integrated virus at producing genomes that are packaged into virions. What accounts for this dichotomy? Apparently uDNA-derived full length RNA is able to associate with the machinery of viral assembly more efficiently than iDNA-derived RNA. We hypothesize that the higher rate of iDNA transcription consumes the local supply of some necessary and limiting factor(s) faster than it/they can be provided, while the lower production rate of uDNA transcription more closely matches the availability of the factor(s). Studies are underway to test this hypothesis.

Such competition for limited cellular resources would not necessarily counteract the type of "beneficial" cooperativity (to the virus) which we have described. On the contrary, by successfully competing with integrated proviruses, "disadvantaged" members of the viral population, i.e. viruses with some functional defect, or viruses that are otherwise functional but which fail to integrate for stochastic reasons, would be able to contribute to the replicating population. The advantage to this would be the preservation of extant genetic diversity, lowering of genetic drift, an increase in effective population size, and the promotion of further diversification and evolution. On theoretical grounds at least, each of these would benefit viral fitness and contribute to viral persistence. There is also evidence that diversity of HIV-1 itself, apart from selective advantages of individual strains which might arise, promotes viral persistence [85,86]. The replication of unintegrated HIV-1 could contribute to such a process. Also within the realm of speculation, sequential infection of cells might favor the viruses which infect after initial productive infection. If a cell already contains Tat, Rev and virion components undergoing assembly, an incoming virus might gain a kinetic advantage by skipping integration. The time required for integration would be subtracted from the time required to complete its replication cycle. Although virus-induced CD4 down modulation can prevent reinfection, it takes a day or two for a cell to be resistant to reinfection, during which time incoming viruses might be at a selective advantage through virus complementation such as we have described.

## Methods

### Viruses

Schematic diagrams of all reporter viruses can be found in Additional File 1: Virus schematics. Fluorescent protein reporter viruses bearing genes for EGFP (GFP), EYFP (YFP) and ECFP (CFP) cloned between the envelope and nef open reading frames have been previously described [43,55]. Nef expression is driven by an IRES element between the reporter gene and nef. All virus constructs are based on HIV-1 NL4-3. DsRed-Express (DsRedX)-containing viruses were constructed in an identical manner using the DsRed-Express gene from Clontech. Replication-competent NLENG1-IRES (containing GFP), NLENY1-IRES (containing YFP), NLENC1-IRES (containing CFP) and NLRX-IRES (containing DsRedX)) and replication-defective viruses containing two stop codons in the envelope reading frame (NLENG1-ES-IRES, NLENY1-ES-IRES, NLENC1-ES-IRES and NLRX-ES-IRES) were constructed for each reporter virus [43]. For simplicity, in this report we designate all GFP viruses as either WT-GFP or D116N-GFP to indicate the status of the integrase gene. Similar nomenclature is used for all viruses. For single round infection assays, envelope defective viruses were pseudotyped with VSV-G protein. Envelope-defective dual reporter viruses were constructed by substituting the region of NL-r-HSAS which contains the murine CD24 (HSA) gene in place of vpr [59] into NLENG1-ES-IRES and NLENG1-ES-IRES-D116N, and are named virus NLHGESI and NLHGESI-D116N for correspondence purposes. In data presentation they are designated WT-GFP/HSA dual reporter and D116N-GFP/HSA dual reporter virus, respectively. The D116N integrase mutation was constructed by recombinant PCR, which introduced a single G to A base pair substitution in the first nucleotide of integrase codon 116, changing it from aspartic acid to asparagine. A translationally silent BamHI restriction site was also introduced 8 bp downstream of the G->A mutation which allows confirmation of cloning by restriction digestion.

HIV-1 virus stocks were generated by transfection of 293T cells as described [43] except that cells were transfected with FuGENE 6 (Roche) or polyethylenimine (PEI) (Sigma) [87]. Infections were typically performed below an MOI of 0.2. An exception is in Figure 5 where up to an MOI of 1.6 of WT virus was employed in order to generate an infection response curve at a wide range of infection rates. When required for PCR, virus stocks were treated with DNase (Invitrogen) at 10 U/ml for 30 minutes at room temperature. VSV-G pseudotyping of envelope-defective viruses was performed by cotransfection of 293T cells with a VSV-G expression plasmid (Clontech) at a HIV/VSV-G plasmid ratio of 8:1. For Vpr complementation of Vpr-negative viruses, an NL4-3 Vpr expression plasmid was cotransfected with NLHGESI (WT-dual reporter virus) or NLHGESI-D116N (D116N-dual reporter virus). For virus transfer assays, 9–18 hours after infection of producer cells, residual input virus was removed by a cell wash in Hanks basal salt solution (HBSS) followed by a 10 minute incubation with 1 mg/ml pronase in HEPES-buffered HBSS for 10 min at room temperature, followed by two washes in complete medium. One day and two days later supernatants were collected, cells and debris removed by centrifugation at 1000 g for 10 minutes, then these supernatants were used to infect Jurkat-Tat cells. Infections were performed in the presence of 10 μg/ml DEAE dextran, or alternatively target cells were infected via spinoculation of virus transfer supernatants by centrifugation at 1200 g for 2 hours at 25°C [88]. Integrase inhibitor 118-D-24 (200 μM), Zidovudine (AZT) and Efavirenz (1 μg/ml each) were applied at the time of infection where indicated. Stock solution of 118-D-24 was prepared in DMSO at 100 mM. Where indicated, Indinavir sulfate (2 μM) was applied to cells within 12 hours of infection. Collection of day 3 viruses was done following a medium change on day 2. All antiviral compounds were obtained from the NIH AIDS Reagent Repository.

### Cells

293T was from the American Type Culture Collection. Jurkat (clone EL-6), and Jurkat-Tat were from the ARRRP. Jurkat-Tat cells were used only after selection in G418 (Sigma) to maintain Tat expression. 293T was maintained in Gibco Advanced (reduced serum requirement) DMEM with 5% fetal bovine serum (FBS) plus penicillin and streptomycin and 50 μM β-mercaptoethanol; Jurkat and Jurkat-Tat were cultured in Gibco Advanced RPMI-1640 with 5% FBS plus penicillin and streptomycin and 50 μM β-mercaptoethanol;. Primary CD4+ T cells were cultured in the same medium as Jurkat except with 10% FBS. Primary CD4+ T cells were generated from healthy HIV-negative donor PBMC isolated by Ficoll-Hypaque centrifugation, followed by negative selection of plastic-adherent cells. CD8+ cells were next removed using anti-CD8 magnetic Dynabeads (Dynal) and the remaining non-adherent cells were stimulated with anti-CD3/CD28 T Cell Expander DynaBeads (Dynal) as per the manufacturer's instructions for 1–3 days prior to infection. IL-2 (50 U/ml) (obtained from the NIH) was added 24 hours after bead stimulation and at every medium change.

### Flow cytometry

Flow cytometry was performed on a Becton-Dickinson FACSort upgraded to contain 488 nm, 407 nm and 637 nm lasers and 5 fluorescence detectors (Cytek Development). GFP and YFP were detected in FL-1 and FL-2 using 505/5 and 550/30 filters, respectively, and a 540SP dichroic splitter. CFP was detected using a 575/15 filter off the 407 nm laser. Compensation was applied during data collection based on single color controls. Data collection was with CellQuest Pro software for the Mac (BD Biosciences). Flow data was analyzed using FlowJo 8 for the Mac (Tree Star). HSA expression was analyzed using APC-conjugated anti-mCD24 antibody, clone M1/69 (BioLegend). Twenty four hours prior to staining, cells were pronase treated as described above to remove any mCD24 (HSA) deposited onto cell membranes by virions during initial infection.

### HIV-1 p24 Gag ELISA

ELISA assays were performed using reagents and protocol kindly provided by Susan Zolla-Pazner, New York University [89]. Briefly, a sandwich ELISA employs 96 well plates coated with anti-p24 human mAb 91–5. Detection of bound p24 is performed using biotinylated anti-p24 human mAb 241D. Plates were developed using a streptavidin-alkaline phosphatase ELISA amplification system (Invitrogen) and read at 490 nm. All samples were run in duplicate with SD <10%.

### Quantitative Real Time PCR for DNA and RNA

#### DNA PCR

DNA was isolated from infected cells using the DNeasy Blood & Tissue kit (Qiagen) and RNase A RNA digestion (Sigma R4642). Quantitative real-time DNA PCR was performed using the QuantiTect Probe PCR kit (Qiagen) and primers and TaqMan probes (Integrated DNA Technologies) specific for GFP [90], DsRed [91] HSA [92] and β-globin [93] that have been described previously. Amplification and detection were performed on a Chromo4 real-time PCR machine (Bio-Rad) and analyzed using the manufacturer's Opticon Monitor software. A reference standard for determination of cell numbers by amplification of β-globin was generated using DNA isolated from uninfected Jurkat cells, quantitated by spectrophotometry and serially diluted (range 1400-1.4 cells/μl, linear range in PCR 1400-14 cells/μl, *r*^2 ^> 0.99). To quantitate relative amounts of GFP, DsRedX and HSA nucleic acids we developed DNA and RNA target sequences carrying one copy of each PCR target, guaranteeing equal stoichiometry of each target for relative quantification of unknowns. To generate this standard, the GFP, DsRed and HSA target DNA sequences were amplified by PCR then linked using recombinant PCR. For RNA production (see below) a T7 promoter was introduced at the 5' end of this sequence, in the order: T7p-GFP-DsRedX-HSA (called "T7GRH standard"), yielding a 279 bp product. For DNA PCR, this PCR product was purified following agarose gel electrophoresis, quantitated by spectrophotometry and serially diluted to generate a standard curve (range 100,000-1 copies/μl, linear range 100,000-10 copies/μl, *r*^2 ^> 0.99).

**qPCR for integrated HIV-1 DNA **was performed using Alu PCR as previously described [13,94] on an ABI 7200 real time PCR machine. Briefly, total nucleic acids from infected cells were extracted in urea lysis buffer then extracted by phenol/chloroform and precipitated with ethanol. Two nested PCR reactions were performed, then first using an Alu forward primer (5'-GCCTCCCAAAGTGCTGGGATG-3') and HIV-1 gag reverse primer (5'-GCTCTCGCACCCATCTCTCTCC-3'). The second PCR amplification employed internal primers and probes SR1 forward primer, M667 reverse primer and ZXF probe previously described [93]. A standard curve representing integrated HIV sequences was generated from cells infected with a nonspreading HIV-based reporter vector. In control experiments, there was no background from nonintegrated viral DNA, and values for integrated DNA copies varied less than 20% within triplicates. The β-globin standard was used to determine the approximate number of proviruses per cell (used the same primer-probe set as in the previous section).

#### RT-PCR

RNA was isolated from virus-containing culture medium or from infected cells using the RNeasy Mini Kit (Qiagen) (including QIAshredder homogenization and on-column DNase treatment). Quantitative real-time reverse transcriptase (RT) PCR was performed using primers and TaqMan probes (Integrated DNA Technologies) specific for GFP [90], DsRed [91] and β-2 microglobulin [95,96], with the QuantiTect Probe RT-PCR kit (Qiagen). No-RT controls were included for each RNA preparation. Amplification and detection were performed on the Chromo4 instrument. For relative quantification of GFP and DsRedX RNA, a T7-promoted transcription reaction was run off the T7GRH standard DNA using the Riboprobe *in vitro *transcription kit (Promega). Residual DNA was eliminated by DNase digestion, then the RNA was purified using the RNeasy Mini Kit, quantitated by spectrophotometry and serially diluted to generate a standard curve (range 10,000,000-1 copies/μl, linear range in RT-PCR was 10,000,000-100 copies/μl, *r*^2 ^> 0.99). RT-PCR on virions: Virus-containing culture supernatants were either directly lysed in RNA extraction buffer for RT-PCR or first RNase treated to remove non-virion-associated RNA. No effect on HIV-1 RNA copy number resulted from prior RNase treatment, while free cellular β-2 microglobulin was typically reduced from about 4 cell equivalents to 2 per 50μL of sample. HIV-1 RNA copy number was ≥1000 fold greater than cellular β-2 microglobulin in culture medium, while cellular extracts contained 20 to >100 fold excess of β-2 microglobulin mRNA vs. HIV-1 RNA, a 5 orders of magnitude shift. These controls demonstrate that PCR amplification was specific for virion-packaged RNA.

## Competing interests

The authors declare that they have no competing interests.

## Authors' contributions

HCG performed PCR analysis of HIV-1 DNA and RNA. DNV and GCB performed PCR analysis for integrated DNA. SAB and SDL performed ELISA assays, virus infections and flow cytometry. DNL conceived and directed this study and wrote the manuscript. All authors have approved the manuscript.

## Supplementary Material

Additional file 1 Reporter virus schematic diagrams. For each figure in the manuscript, schematic diagrams are presented of the reporter viruses employed, showing the HIV-1 open reading frames and the locations of the reporter genes inserted. Click here for file
