# Supplementary material for: Viral complementation allows HIV-1 replication without integration
Source: Retrovirology. 2008 Jul 9;5:60. doi: 10.1186/1742-4690-5-60 (PMC2474848; doi:10.1186/1742-4690-5-60)
Supplement: Additional file 1 — Reporter virus schematic diagrams. For each figure in the manuscript, schematic diagrams are presented of the reporter viruses employed, showing the HIV-1 open reading frames and the locations of the reporter genes inserted. [file 1742-4690-5-60-S1.pdf]

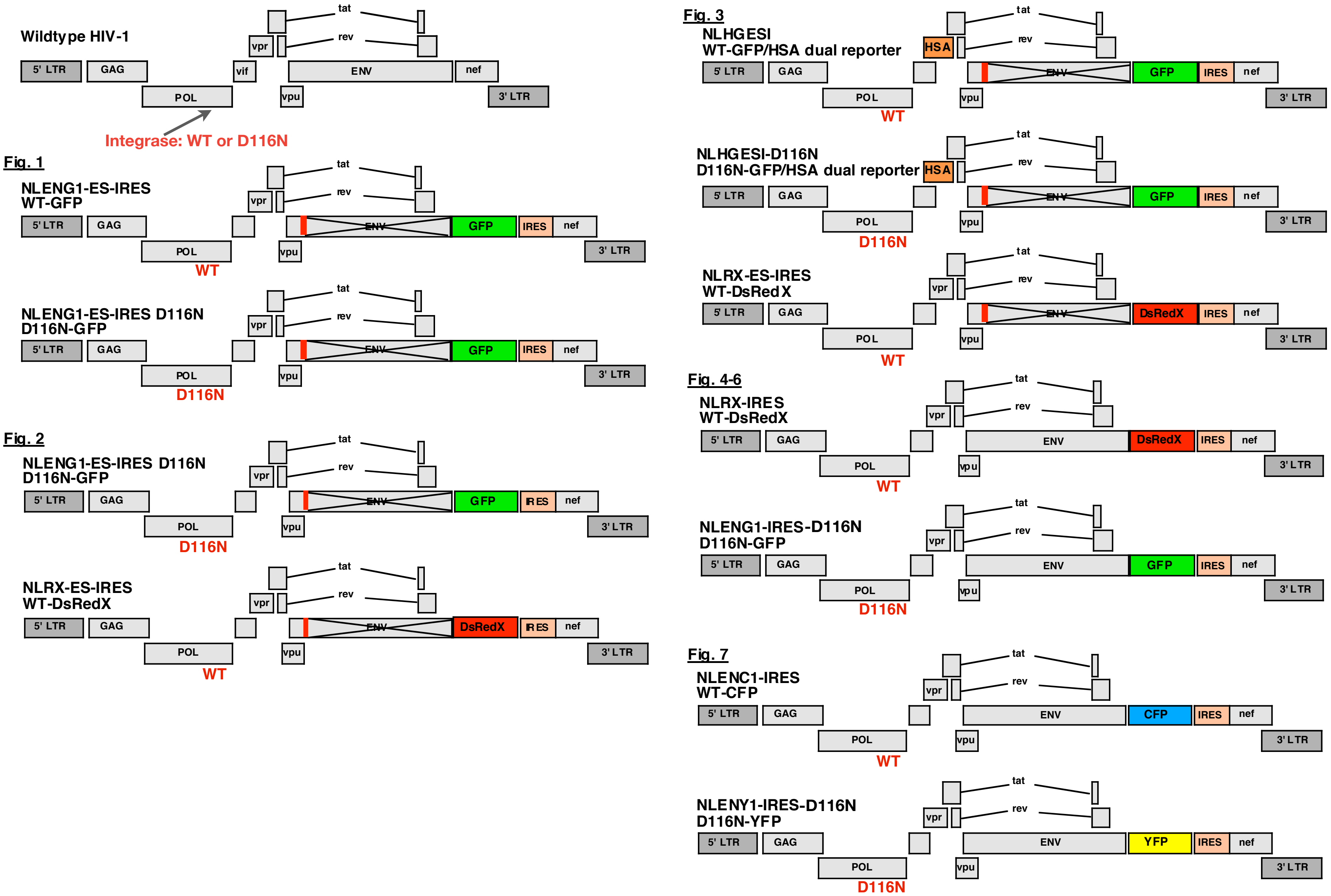

Wildtype HIV-1
5' LTR
GAG
POL
vif
vpr
tat
rev
vpu
ENV
nef
3' LTR
Integrase: WT or D116N
Fig. 1
NLENG1-ES-IRES
WT-GFP
GFP
IRES
WT
NLENG1-ES-IRES D116N
D116N-GFP
D116N
Fig. 2
NLENG1-ES-IRES D116N
D116N-GFP
NLRX-ES-IRES
WT-DsRedX
DsRedX
Fig. 3
NLHGESI
WT-GFP/HSA dual reporter
HSA
NLHGESI-D116N
D116N-GFP/HSA dual reporter
NLRX-ES-IRES
WT-DsRedX
Fig. 4-6
NLRX-IRES
WT-DsRedX
NLENG1-IRES-D116N
D116N-GFP
Fig. 7
NLENC1-IRES
WT-CFP
CFP
NLENY1-IRES-D116N
D116N-YFP
YFP

Gelderblom et al. Additional File 1: Virus Schematics.

Schematic representation of reporter viruses employed in this study. Viruses containing GFP, YFP and CFP genes after the env gene have been previously described in references 43 and 55. For this study additional viruses were constructed containing DsRed-Express (DsRedX) cloned after the env gene identically to the previously described viruses, designated NLRX-ES-IRES and NLRX-IRES. Integrase D116N variants were constructed as described in the methods section of the present work. GFP/HSA dual reporter viruses NLHGESI and NLHGESI-D116N were constructed by merging the HSA reporter viruses NL-r-HSAS from reference 59 with NLENG1-ES-IRES and NLENG1-ES-IRES D116N. Envelope function was eliminated from the "ES" series of viruses by insertion of two stop codons in the env gene after the vpu open reading frame. For clarity in the manuscript, viruses are designated by descriptive names WT-DsRed, D116N-GFP, etc. rather than the longer full names. Plasmid requests should use the NLENG1, or NLRX or NLHG virus terminology.
